# Development and maturation of the fibrous components of the arterial roots in the mouse heart

**DOI:** 10.1111/joa.12713

**Published:** 2017-10-15

**Authors:** Rachel Richardson, Lorraine Eley, Charlotte Donald‐Wilson, Jonathon Davis, Natasha Curley, Ahlam Alqahtani, Lindsay Murphy, Robert H. Anderson, Deborah J. Henderson, Bill Chaudhry

**Affiliations:** ^1^ Cardiovascular Research Centre Institute of Genetic Medicine Newcastle University Newcastle upon Tyne UK

**Keywords:** aortic root, arterial wall, bicuspid aortic valve, fibrous tissue, hypoplastic left heart syndrome, interleaflet triangles, neural crest cells, second heart field, sinus, smooth muscle cells, valves

## Abstract

The arterial roots are important transitional regions of the heart, connecting the intrapericardial components of the aortic and pulmonary trunks with their ventricular outlets. They house the arterial (semilunar) valves and, in the case of the aorta, are the points of coronary arterial attachment. Moreover, because of the semilunar attachments of the valve leaflets, the arterial roots span the anatomic ventriculo‐arterial junction. By virtue of this arrangement, the interleaflet triangles, despite being fibrous, are found on the ventricular aspect of the root and located within the left ventricular cavity. Malformations and diseases of the aortic root are common and serious. Despite the mouse being the animal model of choice for studying cardiac development, few studies have examined the structure of their arterial roots. As a consequence, our understanding of their formation and maturation is incomplete. We set out to clarify the anatomical and histological features of the mouse arterial roots, particularly focusing on their walls and the points of attachment of the valve leaflets. We then sought to determine the embryonic lineage relationships between these tissues, as a forerunner to understanding how they form and mature over time. Using histological stains and immunohistochemistry, we show that the walls of the mouse arterial roots show a gradual transition, with smooth muscle cells (SMC) forming the bulk of wall at the most distal points of attachments of the valve leaflets, while being entirely fibrous at their base. Although the interleaflet triangles lie within the ventricular chambers, we show that they are histologically indistinguishable from the arterial sinus walls until the end of gestation. Differences become apparent after birth, and are only completed by postnatal day 21. Using *Cre‐lox*‐based lineage tracing technology to label progenitor populations, we show that the SMC and fibrous tissue within the walls of the mature arterial roots share a common origin from the second heart field (SHF) and exclude trans‐differentiation of myocardium as a source for the interleaflet triangle fibrous tissues. Moreover, we show that the attachment points of the leaflets to the walls, like the leaflets themselves, are derived from the outflow cushions, having contributions from both SHF‐derived endothelial cells and neural crest cells. Our data thus show that the arterial roots in the mouse heart are similar to the features described in the human heart. They provide a framework for understanding complex lesions and diseases affecting the aortic root.

## Introduction

**Figure 1:**
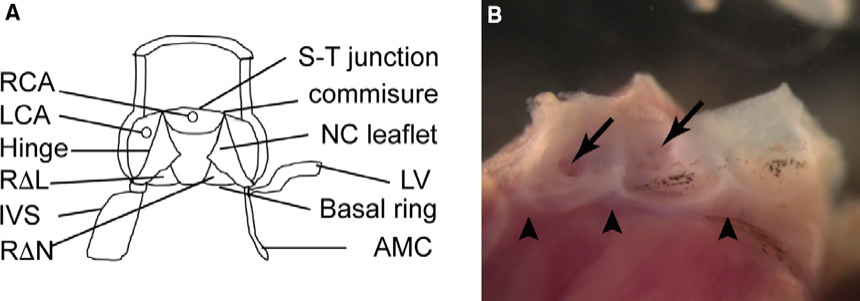
Basic anatomy and nomenclature used for aortic root. (A) Cartoon showing basic structure of the mature aortic root and the nomenclature used. (B) View of the opened aortic root from an adult mouse heart. The valve leaflets have been removed to display the sinus walls and the coronary orifices (arrows). Above the points of attachment of the valves the wall is arterial, whereas below it is ventricular muscle. The interleaflet triangles (arrowheads) that lie between the curved points of attachment of the valve leaflets can be seen. AMC, aortic‐mitral continuity; IVS, interventricular septum; LCA, left coronary artery; LV, left ventricular myocardium; NC leaflet, non‐coronary leaflet; RCA, right coronary artery; RΔL, right‐left interleaflet triangle; RΔN, right‐non‐coronary interleaflet triangle; S‐T junction, sinutubular junction.

The arterial roots connect the ventricular outflow tracts to the intrapericardial components of the ascending aorta and pulmonary trunk (Fig. 1). They house the aortic and pulmonary valve complexes, and are an important site of congenital malformation and pathology in adult life. Congenital abnormalities of the ascending aorta and its root are commonly associated with intra‐cardiac malformations, such as tetralogy of Fallot, or abnormalities of the aortic arch. For example, severe aortic stenosis (narrowing) or atresia (blockage) are principle features of hypoplastic left heart syndrome, in which the left ventricle is also hypoplastic (Tchervenkov et al. 2006). Similarly, dilation and calcification of the aortic root frequently accompany bicuspid aortic valve (Siu & Silversides, 2010), which represents one of the most common causes of cardiac intervention. It has been suggested that the associations between abnormalities of the arterial roots and other cardiovascular anomalies have a haemodynamic aetiology. For example, abnormalities in blood flow secondary to a bicuspid valve have been suggested to lead to aneurysm or calcification (Siu & Silversides, 2010). A genetic basis for these malformations, however, is becoming increasingly apparent (Siu & Silversides, 2010). An alternative explanation is that a disturbance of development in a common progenitor cell type links these pathologies. Indeed, the late failure of the transposed pulmonary homograft after the Ross procedure, where the pulmonary root is grafted into the aortic position to treat severe aortic stenosis, may indicate a pathological process common to both arterial roots (David et al. 2000).

The aortic and pulmonary roots are complex, made up of valve leaflets, their supporting sinuses, and the fibrous interleaflet triangles (Fig. 1A,B). Although a significant amount of research has focused on the formation of the valve leaflets, little has focused on the development, or maldevelopment, of the remainder of the valve complex. Indeed, it is unclear to what extent abnormalities in the formation of the supporting structures of the leaflets – the sinus walls and points of attachment of the leaflets to the walls – can lead to pathologies such as aortic stenosis. Unlike the free‐standing pulmonary root, which is supported entirely by ventricular myocardium, parts of two of the aortic leaflets are in fibrous continuity with the leaflets of the mitral valve (Loukas et al. 2014), with part of the root also in connection with the membranous part of the ventricular septum. Surprisingly little is known about the cellular origins and processes involved in the development of the supporting structures of the arterial roots. For example, although a small number of studies have elucidated the progenitors of the ascending arterial trunks (Hutson & Kirby, 2007; Vincent & Buckingham, 2010; Harmon & Nakano, 2013), only a limited number of studies, carried out during the last century, have focused on the complexity of the tissue relationships and architecture of the arterial roots (Jackson et al. 1995; Ya et al. 1998; Qayyum et al. 2001). These existing studies were also based on the erroneous concept of a pre‐segmented heart tube. Reagents were not available at that time to identify and trace the lineages of these different multipotent cell populations. Thus, it was not possible to establish the formation of the laminar components of the proximal aorta and aortic root, nor whether the fibrous tissues in the roots arise from regression or transformation of previously existing myocardial tissues.

In this study, we use *Cre‐lox* technology to identify the progenitors that form the walls of the aortic and pulmonary roots, showing how these cells differentiate and mature. We show that, whereas the fibrous attachments of the valve leaflets are derivatives of the outflow cushions, and thus have contributions from both NCC and SHF‐derived endothelial cells, the fibroblasts in the walls of the valvar sinuses share a precursor with the SMC in this region. They are derived almost entirely from the SHF, without passing through the endothelial lineage. Here, we clarify the formation of the arterial roots and their adjoining arterial walls. This data will have relevance for understanding both congenital and adult pathologies, and will be needed to interpret genomic analyses of these disease‐prone segments of the heart.

## Materials and methods

### Mouse strains and histological analysis

*Mef2c‐AHF‐Cre, Wnt1‐Cre, Nkx2‐5‐Cre, Isl1‐Cre*,* Mlc2v‐Cre*,* WT1‐ERT‐Cre* and *Tie2‐Cre* (Chen et al. 1998; Danielian et al. 1998; Kisanuki et al. 2001; Moses et al. 2001; Verzi et al. 2005; Yang et al. 2006; Zhou et al. 2008) mice, intercrossed with the *ROSA‐Stop‐eYFP* (Srinivas et al. 2001) or *mTmG* (Muzumdar et al. 2007) line, were used to follow cells of the required lineage/cell type. Timed matings were carried out overnight, with the presence of a copulation plug designated embryonic day (E) 0.5. Littermate controls were used where appropriate. Mice were maintained according to the Animals (Scientific Procedures) Act 1986, United Kingdom, under project licence PPL 30/3876. All experiments were approved by the Newcastle University Ethical Review Panel. Embryos and dissected hearts from postnatal animals were harvested at different developmental stages, rinsed in ice‐cold phosphate‐buffered saline (PBS) and fixed overnight in 4% paraformaldehyde before paraffin‐embedding. For routine histological analysis, paraffin‐embedded embryos or isolated hearts were sectioned and stained with haemotoxylin and eosin, Masson's trichrome or Miller's elastin, following standard protocols.

### Immunofluorescence

Embryos/hearts were rinsed in ice‐cold PBS and fixed overnight in 4% paraformaldehyde before paraffin‐embedding (Boczonadi et al. 2014; Ramsbottom et al. 2014). Sections were cut at 8 μm using a rotary microtome (Leica). Slides were de‐waxed with Histoclear and rehydrated through a series of ethanol washes. Following washes in PBS, antigen retrieval was performed by boiling slides in citrate buffer (0.01 mol L^−1^) pH 6.3 for 5 min. Samples were blocked in 10% fetal calf serum (FCS) and then incubated overnight at 4 °C with the following antibodies: cTnI (HyTest), Fsp1 (Millipore), GFP, alpha smooth muscle actin, Collagen I, SM22 alpha, Sox9, Periostin (Abcam). For immunofluorescence, samples were incubated at room temperature for 1 h, with secondary antibodies conjugated to either Alexa 488 or Alexa 594 (Life Technologies). Fluorescent slides were then mounted with Vectashield Mounting medium with DAPI (Vector Labs). For non‐fluorescent staining, samples were incubated with biotinylated secondary antibodies for 1 h, and then with AB complex (Vector labs) for a further hour before being stained with DAB and mounted using Histomount. Immunofluorescence images were collected using a Zeiss Axioimager Z1 fluorescence microscope equipped with a Zeiss Apotome 2 (Zeiss, Germany). Acquired images were processed with axiovision rel 4.9 software (Zeiss). Bright field images were captured using the Zeiss Axioplan (Zeiss).

## Results

### Structure of the mouse arterial roots

**Figure 2:**
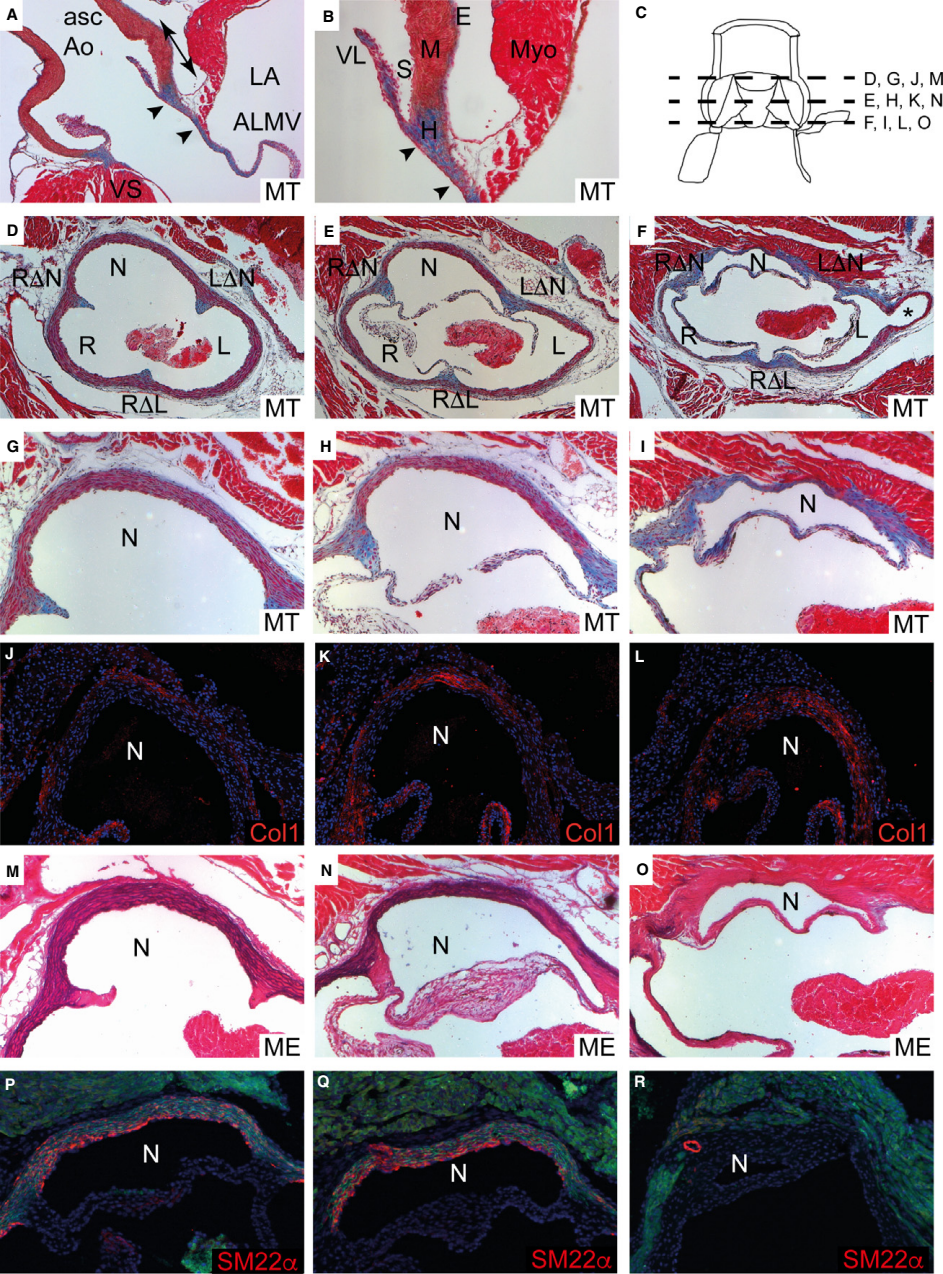
Histological analysis of the mature aortic valve sinus walls. (A,B) Longitudinal sections through the aortic root showing stained with Masson's trichrome showing muscular (red) and fibrous (blue) components. The double‐headed arrow in (A) indicates the extent of the root. The aortic‐mitral continuity is delineated by arrowheads. The three layers of the arterial wall can be seen in higher magnification (B). (C) Cartoon illustrating the level of sections in (D–R). (D–R) Transverse sections through the distal (sinutubular junction; D, G, J, M, P), middle (E, H, K, N, Q), and proximal (basal; F, I, L, O, R) levels of the aortic root stained with Masson's trichrome (D–I), Collagen‐I antibody (J–L; red), Miller's elastin (M–O) and SM22α antibody (P–R; red). The most distal parts of the valve attachment (the commissures) can be seen (in D, G, M) in continuity with the corresponding interleaflet triangle. Masson's trichrome staining shows that distally the media of the sinus wall is largely composed of red SMC (D, E, G, H) with only a thin layer of blue fibrous tissue, but this balance changes moving proximally through the root, such that at the most basal level of the valve sinuses, the wall is entirely stained blue for fibrous tissue (F, I). Collagen‐I immunostaining (J–L) matches the fibrous staining with Masson's trichrome with little staining at the distal part of the root (J), but the whole wall stained proximally (L). Miller's elastin staining (M–O) shows that elastic laminae (black) are abundant in the distal and mid regions of the aortic root (M, N), but at the most basal level of the aortic root the sinus walls have few, if any, elastic laminae (O). Immuno‐staining for SM22α confirms this distribution of SMC, with abundant staining (red) in the distal and mid‐regions of the sinus walls (P, Q) but none at basal levels (R). Cardiomyocytes and elastic fibres autofluoresce and appear green in these images. *Coronary artery. ALMV, aortic leaflet of the mitral valve; Asc Ao, ascending aorta; E, epicardium; L, left sinus (also R and N for others); LΔN, left‐non coronary interleaflet triangle (also RΔN and RΔL for others); LA, left atrium; M, media; ME, Miller's elastin; MT, Masson's trichrome; Myo, myocardium; S, sinus; V, ventricle; VL, valve leaflet; VS, interventricular septum.

Histological analysis of the juvenile mouse heart at postnatal day (P) 21 demonstrated that the arterial valve leaflets formed the proximal boundary of the arterial components of the outflow tracts, although they were supported proximally by the myocardial ventricular walls and associated septal structures (Figs 1B and 2A). Although the pulmonary root was also examined, we focus our comments on the aortic root, and describe the pulmonary root only where it differs. Close examination of the wall of the intrapericardial aorta in sections stained with Masson's trichrome, which shows muscle as pink and fibrous tissue as blue, revealed its tri‐layered structure, with an inner endothelial layer (tunica intima) a thick layer of smooth muscle cells (tunica media), coated externally by a layer of fibrous tissue (Fig. 2B). Within the confines of the pericardial reflections, this outer fibrous layer is considered to be epicardium, but it was confluent with the layer overlying the aorta and pulmonary arteries within the mediastinum, where it is termed the tunica adventitia. Transverse sections through the entire aortic root at P21 (see cartoon – Fig. 2C), stained with Masson's trichrome (Fig. 2D–I), an antibody raised against Collagen I (Fig. 2J–L), Miller's elastin (black staining in Fig. 2M–O) and with an antibody raised against SM22α, an SMC‐specific protein (Fig. 2P–R), showed that there was a gradual transition within the valvar sinus walls such that distally, at the tips of the so‐called commissures at the sinutubular junction, they were composed almost entirely of SMC, with only a thin layer of surrounding fibrous tissue (Fig. 2G,J,M,P). In contrast, the walls were entirely fibrous proximally at the base of the leaflets (Fig. 2I,L,O,R).

**Figure 3:**
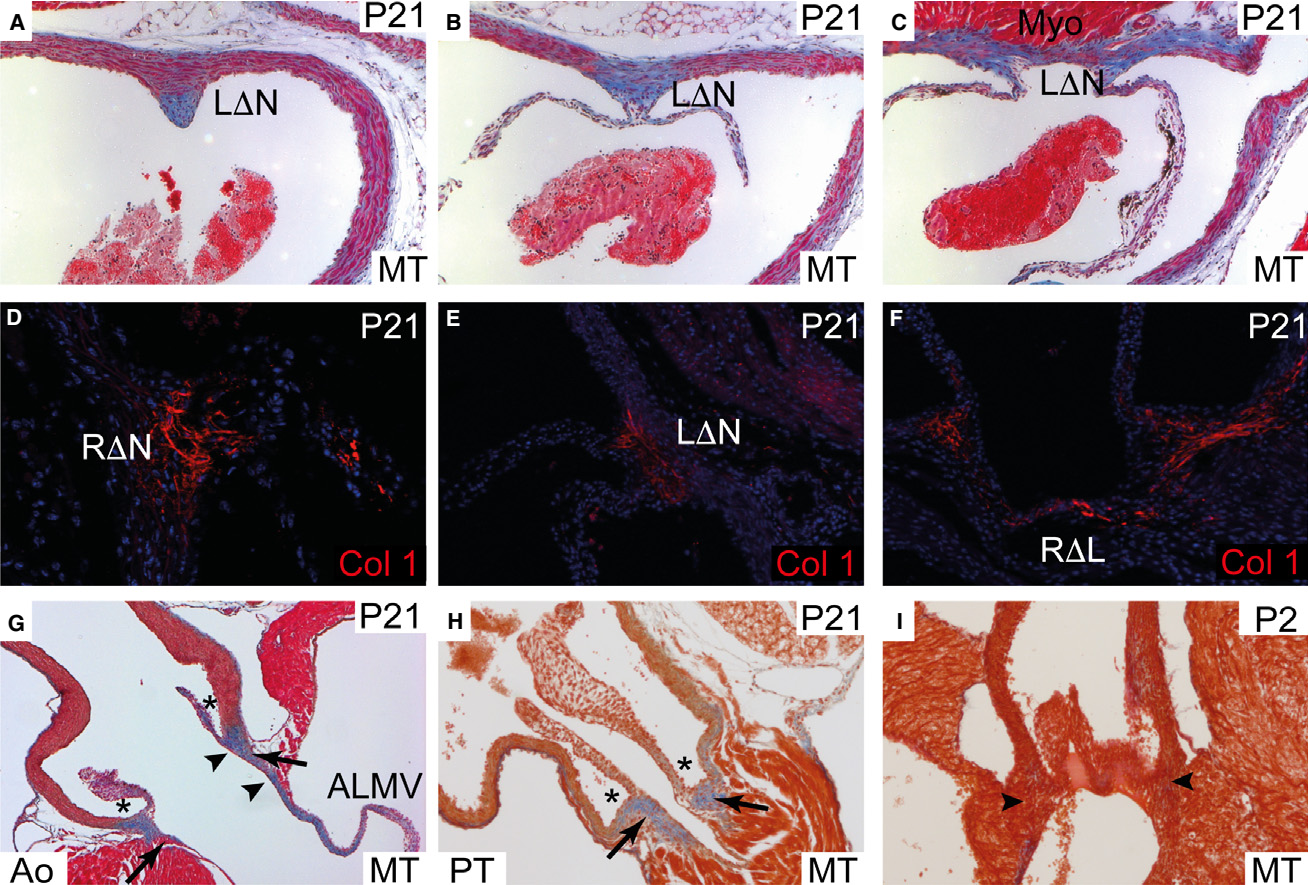
Histological analysis of the mature aortic interleaflet triangles. Unless otherwise stated, all sections are of the aortic root at P21. Sections at the level of the sinutubular junction (A,D), midway through the root (B, E) and at the basal level (C, F). (A–C) Masson's trichrome staining showing that at the most distal level of the root, only the commissure is fibrous (stained blue), whereas the adjacent arterial wall is not. More proximally, the interleaflet triangles between the valve sinuses are fibrous. (D–F) Collagen‐I staining confirms that each of the interleaflet triangles are collagen‐rich. (G, H) Direct comparisons between the aortic (G) and pulmonary roots (H), in longitudinal section, show that although they are very similar in structure, whereas the aortic root is in fibrous continuity with the mitral valve via the aortic‐mitral continuity, the pulmonary root is entirely supported by myocardium. Arrows point to the fibrous valve hinges and arrowheads (in G) delineate the aortic‐mitral continuity. (I) At P2, there is little staining of fibrous tissue in the valve hinges (arrowheads) by Masson's trichrome, suggesting that this may develop postnatally. AMC, aortic‐mitral continuity; Col‐I, collagen‐I; LΔN, left‐non coronary interleaflet triangle (also RΔN and RΔL for others); MT, Masson's trichrome; Myo, myocardium; PT, pulmonary root. *mark the valve sinuses.

On the ventricular side of the mature (P21) root, the interleaflet triangles, interposed between the semilunar hinges as they ascended to the sinutubular junction, were almost exclusively fibrous, as revealed by Masson's trichrome staining (Fig. 3A–C). Immuno‐labelling showed them to be rich in collagen‐I (Fig. 3D–F). The right/left interleaflet triangle (RΔL) sat above the myocardium of the ventricular septum, whereas the right/non‐adjacent triangle (RΔN) was contiguous proximally with the membranous septum. We use ‘non‐adjacent’ in this context because in rare cases of congenitally malformed hearts, the sinus that is furthest from the pulmonary root can give rise to a coronary artery. In this setting, it would obviously be incorrect to describe the sinus as being ‘non‐coronary’. The left/non‐adjacent triangle (LΔN) was incorporated into the area of fibrous continuity with the aortic, or anterior, leaflet of the mitral valve (Fig. 3G). As with the aorta, the pulmonary root contained fibrous interleaflet triangles rich in collagen‐I. In contrast to the aortic root, all of the interleaflet triangles of the pulmonary root were supported at their base by myocardium of the ventricular infundibulum (Fig. 3H). In the aortic root, myocardium supports the parts of the two sinuses that usually give rise to the coronary arteries. The entirety of the proximal part of the sinus walls of the aorta, nonetheless, was fibrous. This fibrous tissue extended out from the base of the valve leaflets into the supporting wall, as the valve hinges (Fig. 3G,H). At the sinutubular junction, however, the fibrous tissue did not extend into the supporting wall (Fig. 3A). Neither the interleaflet triangles, nor the valve hinges, stained for collagen when using Masson's stain trichrome at P2 (Fig. 3I), suggesting that these structures become fibrous only after birth. This observation prompted our further investigation into the cellular origins and timing of this process of differentiation.

### The fibrous tissues within the arterial walls become distinct postnatally

**Figure 4:**
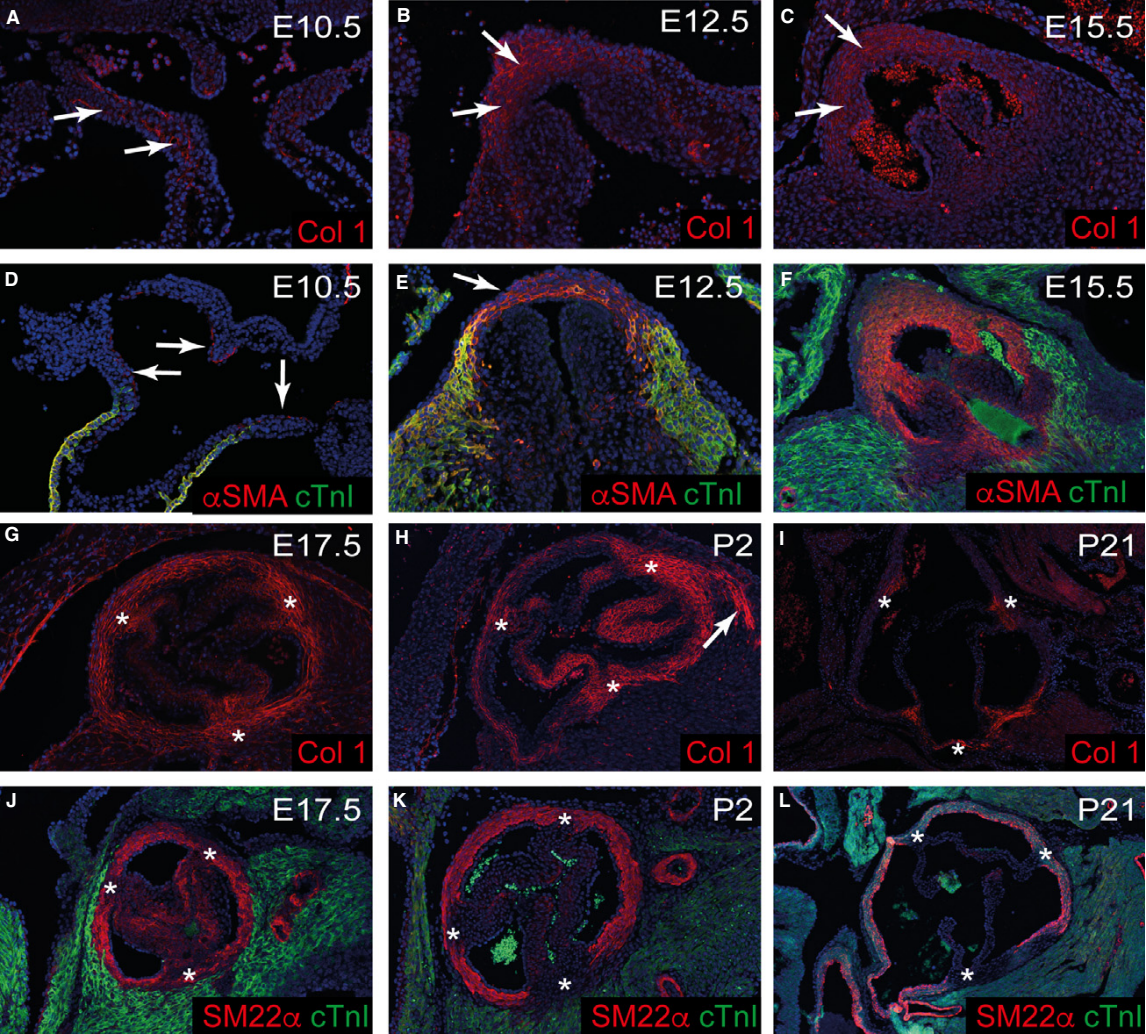
Maturation of the fibrous and SMC walls of the aortic root. Images are of the unseptated outflow tract (A, D) or the aortic root. (A–C, G–I). Collagen‐I (red) is expressed in the non‐myocardial part of the distal outflow tract and aortic sac at E10.5 (arrows in A) and this is maintained at E12.5–E17.5 (arrows in B, C, and in G) in the septated arterial parts of the outflow vessels. By P2, collagen‐I is restricted to the outer part of the media and the interleaflet triangles (* in H), and by P21, is largely restricted to the interleaflet triangles (* in I). Collagen‐I is also expressed in the aortic to mitral fibrous continuity (arrow in H). (D–F, J–L) SMC markers αSMA and SM22α (red) are restricted to the luminal side of the outflow vessel wall at E10.5 and E12.5 (D, E) although by E15.5 (E), they fill the media of the arterialised vessels. The outflow cushions are also restricted to the myocardial part of the outflow tract at E10.5. cTnI (green) is restricted to cardiomyocytes within the proximal regions of the developing outflow tracts at all stages. By E17.5, SM22α is lost from the developing interleaflet triangles (* in J) and this is maintained at P2 and P21 (* in K, L).

Having shown that the fibrous tissues of the heart are incompletely and developed in the immediate postnatal period, we wanted to establish whether the cells producing smooth muscle and fibrous tissue are distinct populations throughout development, or only become distinct from one another after birth. To this end, we used fibrous (collagen‐I) and SMC (SM22α/αSMA) markers, comparing their patterns of expression from E10.5, when the outflow tract is still unseptated, to P21, when the walls of the aorta and pulmonary trunk are mature. At E10.5, collagen‐I was expressed broadly, although at low level, in the distal outflow walls (Fig. 4A). This pattern was maintained at E12.5 and E15.5 (Fig. 4B,C), although at these stages, collagen‐I was more abundant. The SMC markers αSMA/SM22α are not specific for SMC during early development, being expressed also by immature outflow tract cardiomyocytes (yellow staining in Fig. 4D,E). These antibodies, nonetheless, identified a thin layer of cells, distinct from forming myocardium, underlying the endocardium of the non‐myocardial distal outflow walls (arrows in Fig. 4D). Temporal analysis indicated that these cells remained separate from myocardial cells, and were destined to become the SMC of the tunica media. At this stage, notably, the outflow cushions, the precursors of the valve leaflets, were exclusively within the myocardial part of the outflow tract (Fig. 4D). From E12.5 to E15.5, αSMA/SM22α was co‐expressed with collagen‐I throughout the maturing walls of the valvar sinuses and the intrapericardial arterial trunks (compare Fig. 4E,F to B,C). Thus, SMC markers were found in the interleaflet triangles during mid‐late gestation, although these structures are entirely fibrous in the mature heart. It was only around the time of birth, at E17.5 and P2, that the expression patterns of fibrous and SMC markers began to become distinct, with αSMA and SM22α reduced in the interleaflet triangles (Fig. 4J,K). These patterns were fully consolidated by P21, by which time they closely resembled the patterns of fibrous and SMC tissue as revealed using Masson's Trichrome and Miller's elastin staining (compare Fig 4I,L with Fig. 2).

### The valve hinges and commissures are derived from the outflow cushions

**Figure 5:**
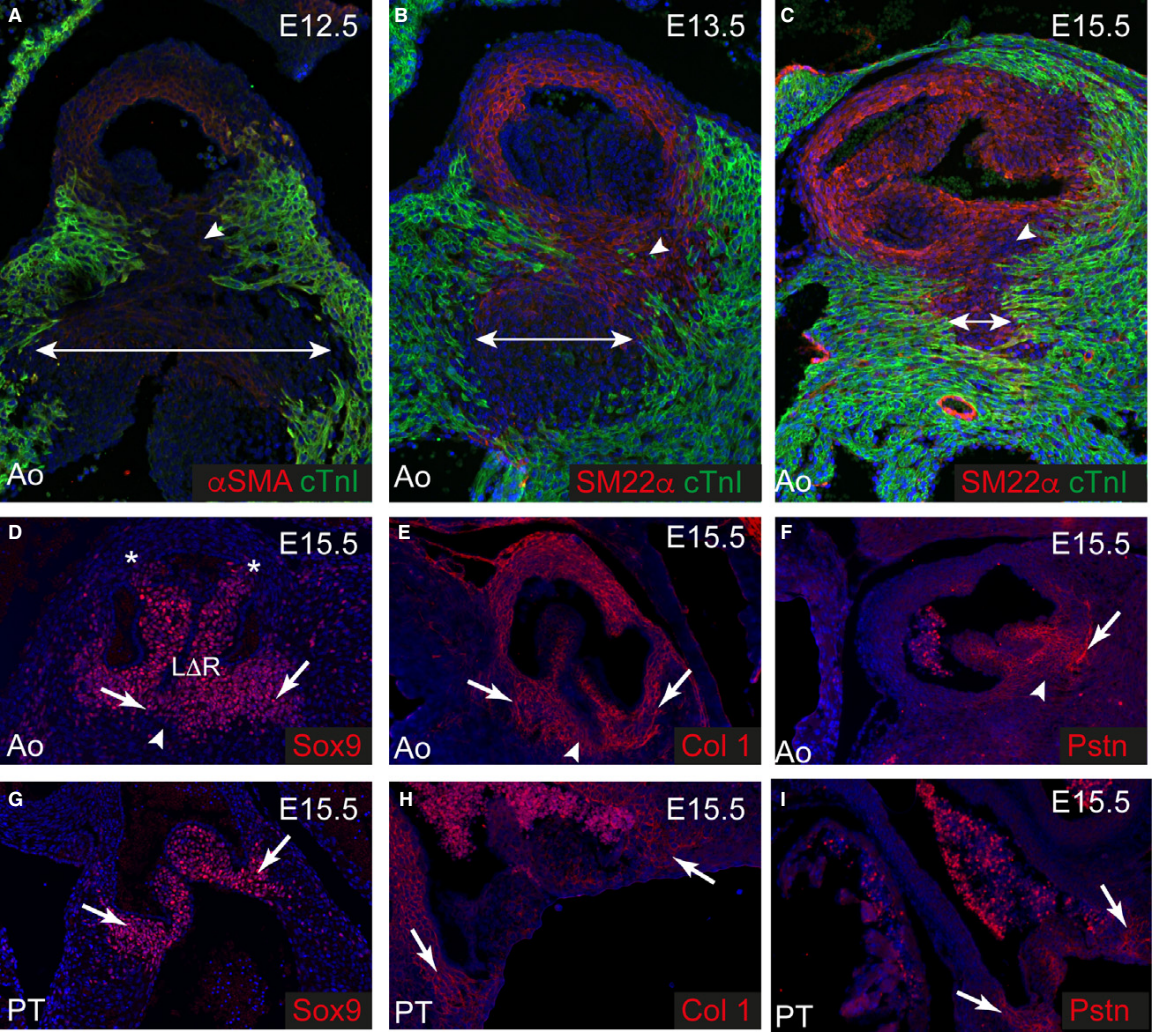
Origins of the valve commissures and hinges from the outflow cushions (A–C). Myocardialisation of the proximal outflow cushions between E12.5 and E15.5 results in the formation of the muscular outlet septum but spares the regions immediately adjacent to the valve leaflets (arrowheads). (D, G) Transverse sections through both the aorta (D) and the pulmonary trunk (G), immuno‐stained for Sox9 (red), show that the valve leaflets and the valve hinges are labelled by Sox9 and thus are derived from the outflow cushions; however, the interleaflet triangles are not (* in D). (E, F, H, I) Collagen‐I (E, H) and periostin (F, I) also label the valve hinges (arrows) in both the aortic and pulmonary roots.

Having established the time course of differentiation of SMC and fibrous tissues at the base of the sinuses and in the interleaflet triangles, we sought to clarify their relationship to the valve hinges and the commissures. These are the structures that attach the valve leaflets to the surrounding wall, which are also fibrous in the mature heart. Specifically, we wanted to establish whether any of these tissues are derivatives of the outflow tract cushions. The right ventricular aspect of the proximal outflow tract cushions is muscularised during mid gestation, between E12.5 and E15.5, thus forming the free‐standing infundibulum by the ingrowth of cardiomyocytes from the outflow tract wall, and concurrent death of the original cushion cells (Fig. 5A–C; Poelmann et al. 1998; van den Hoff et al. 2001; Phillips et al. 2005). This ingrowth of cells leaves a region adjacent to the leaflets that remains non‐muscularised, which corresponds to the valve hinges (arrowhead in Fig. 5A–C). To investigate the characteristics of this tissue, we examined the expression of Sox9, a transcription factor known to be expressed by both the atrioventricular and outflow tract cushions, and the leaflets derived from them, throughout development and into the neonatal period (Akiyama et al. 2004). Analysis at E15.5 showed that the commissures, representing the distal points of attachment of the leaflets with the arterial wall at the sinutubular junction, expressed Sox9, but that this expression did not extend into the arterial wall (* in Fig. 5D). Moreover, the RΔN and LΔN interleaflet triangles did not express Sox9, showing that they are not derived from the outflow cushions. In contrast, the RΔL interleaflet triangle did express Sox9 (arrowhead in Fig. 5D) and also expressed the fibrous markers collagen‐I and periostin (Fig. 5E,F). This difference can be explained by the origin of the RΔL interleaflet triangle from the fused main outflow cushions. At the basal level, the valve hinges also expressed Sox9, but this expression extended into the supporting tissues (arrows in Fig. 5D,G), specifically into the region that remains non‐muscular following myocardialisation of the surface of the proximal cushions. These hinges also expressed collagen I and periostin, typical markers of fibrous tissue (Fig. 5H,I). This supports the idea that the anchoring tissues of the valve hinges are derived from the outflow cushions, and are embedded within the ventricular myocardium that is derived from myocardialisation of the proximal outflow tract cushions, along with the myocardium supporting the aortic root that is transferred to the developing left ventricle. Whereas the myocardialised outflow cushions, along with the initial subaortic outflow myocardium, provide a muscular base for the adjacent leaflets, the anchoring structures of the valve hinges, although also derived from the proximal outflow cushions, retain greater similarities with the leaflets with which they are in continuity (Fig. 5D,G).

### The walls of the arterial roots are derived from NCC and SHF‐derived cells

**Figure 6:**
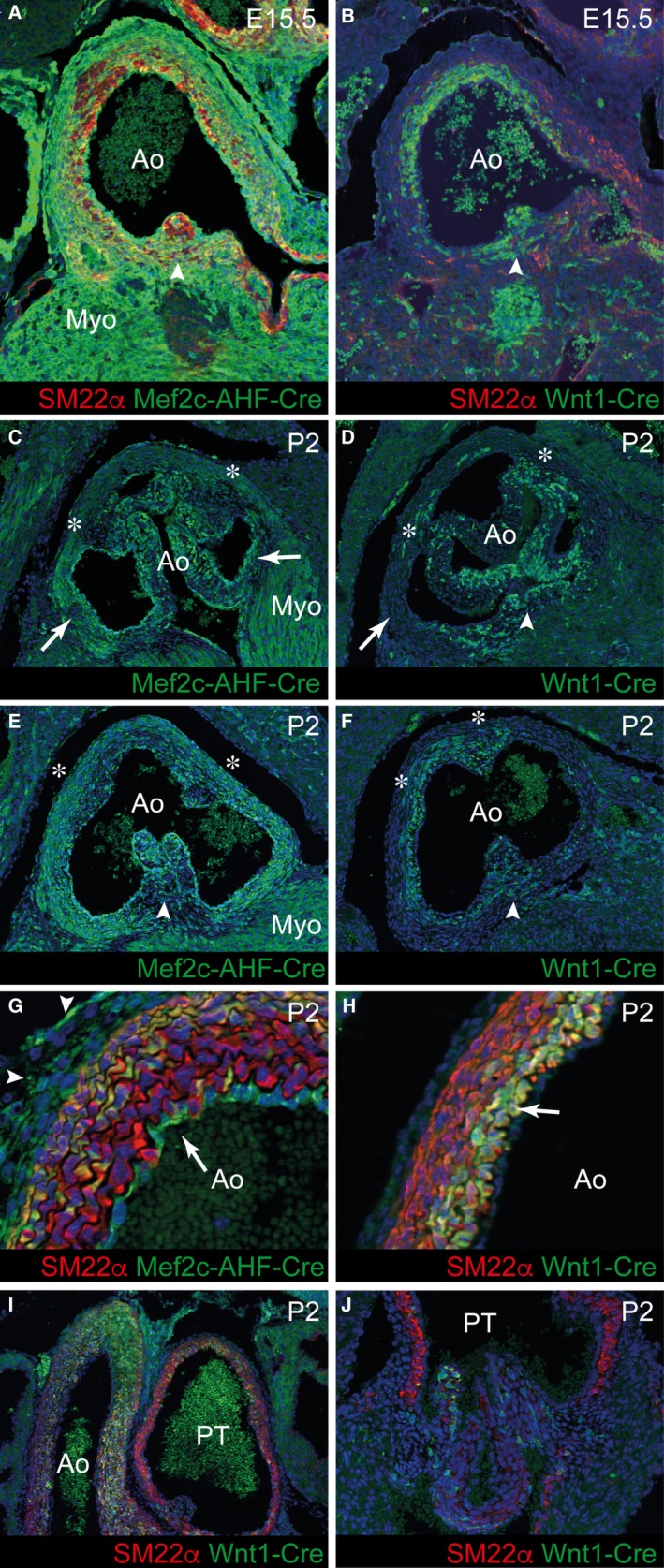
The arterial roots are derived from SHF and NCC. (A, C, E, G) At E15.5 and P2, *Mef2c‐AHF‐Cre* labelled cells localise mainly to the outer part of the wall, including the RΔN and LΔN interleaflet triangles (asterisks in C, E), where they co‐express SM22α(Γ). (B, D, F, H) At the same stages, *Wnt1‐Cre* labelled cells (co‐expressing SM22α in H) are localised mainly to the luminal side of the wall in the distal part of the root and contribute to the inner part of the RΔN and LΔN interleaflet triangles (asterisks in D, F) and commissures. *Wnt1‐Cre*‐labelled cells make a greater contribution to the LΔR interleaflet triangle (arrowheads in B, D, F, *cf*. E). Both *Mef2c‐AHF‐Cre*‐ and *Wnt1‐Cre*‐labelled cells are found in the valve leaflets. (I, J) In comparison with the aortic root, there are fewer *Wnt1‐Cre*‐labelled cells in the walls of the pulmonary root. Ao, aortic root; Myo, myocardium; PT, pulmonary root.

**Figure 7:**
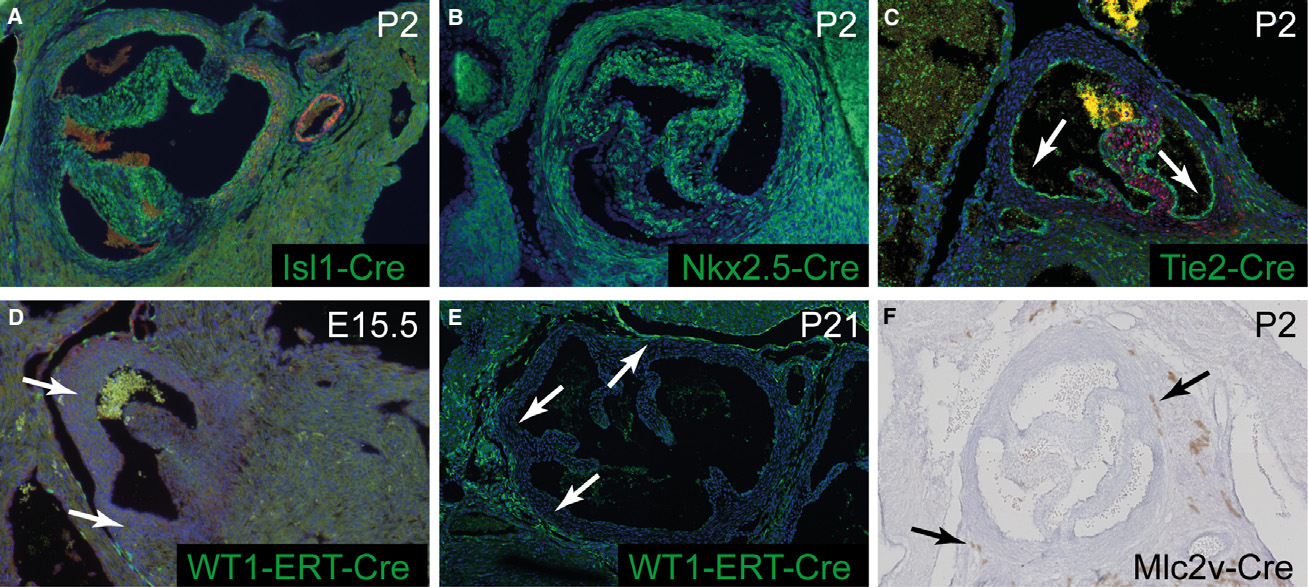
No contributions from endocardial, myocardial and epicardial‐derived cells to the aortic root. (A, B) *Isl1Cre* and *Nkx2‐5‐Cre* patterns are very similar to each other and to *Mef2c‐AHF‐Cre* (see Fig. 4) at P2. (C) *Tie2‐Cre* labels the endocardium (arrows) but not SMC in the media. (D, E) *WT1‐ERT‐Cre* labels cells in the epicardium but not in the media (arrows) at E15.5 and P21. (F) *Mlc2v‐Cre* (brown) gives patchy staining in the myocardium, in some cases very close to the arterial wall (arrows), but is never found in cells within the arterial component of the aortic root including the interleaflet triangles.

It is not known whether the walls of the aortic roots, including both the interleaflet triangles and the basal components of the sinuses, form directly into fibrous tissue, or by trans‐differentiation from SMC or myocardium. To address this we utilised *Cre‐lox* lineage tracing to identify the embryonic origins of the cells that form the arterial roots. All of the myocardial tissues supporting the arterial roots were shown to be derived from the SHF at both E15.5 and P2 (Fig. 6A,C,E). Lineage tracing with *Mef2c‐AHF‐Cre*,* Isl1‐Cre* and *Nkx2‐5‐Cre,* all of which have been used as lineage markers for SHF precursors (Verzi et al. 2005; Yang et al. 2006; Harmon & Nakano, 2013; Figs 6A,C,E and 7A,B), revealed that the cells in the developing sinus walls (arrows in Fig. 6C), which will become fibrous, were also of SHF origin. Distal to the sinutubular junctions, only the SMC in the outer media and the enveloping epicardial layer were SHF derivatives (Fig. 6E,G). In contrast, at the base of the sinuses, *Mef2c‐AHF‐Cre*‐expressing cells were found throughout the wall (Fig. 6C). The intimal (endothelial) layer was labelled not only with *Mef2c‐AHF‐Cre* (arrow in Fig. 6G), but also with *Tie2‐Cre* (Fig. 7C), indicating its nature as SHF‐derived endothelium. This distribution of SHF tissue was complemented by *Wnt1‐Cre* lineage tracing, which defined the remaining tissues in the sinus walls to be of NCC origin. Hence, NCC provided the tissues making up the inner luminal layers of SMC at the level of the sinutubular junction (Fig. 6B,F,H) that were not labelled by *Mef2c‐AHF‐Cre* (compare Fig. 6H with G). They made little, if any, contribution to the basal part of the sinuses (arrow in Fig. 6D). Thus, the SHF and NCC contributions to the sinus walls were complementary, with the SHF providing the fibrous tissues at their base, but both lineages providing SMC more distally. The pulmonary root had a similar composition, although there was a reduced contribution from NCC in comparison with the aortic root (Fig. 6I,J). We were able to exclude transdifferentiation of endothelial cells or epicardially derived cells into the SMC or fibrous tissues of the aortic or pulmonary roots using *Tie2‐Cre* and *WT1‐ERT‐Cre*, respectively (Fig. 7C–E). Similarly, there was no labelling within either the SMC or fibrous parts of the arterial roots in mice containing *Mlc2v‐Cre*‐driven reporter (Fig. 7F). Thus, we could also exclude trans‐differentiation from ventricular cardiomyocytes into fibrous tissues at the proximal parts of the aortic and pulmonary roots. These analyses show that the fibrous tissue forming the proximal components of the valve sinuses are derived almost entirely from the SHF, whereas more distally, towards the sinutubular junctions, the SMC walls are composed of both NCC and the SHF, with the two progenitor lineages being spatially restricted.

Both SHF and NCC contribute to the RΔN and LΔN, with their cellular compositions and distributions matching those of their adjacent sinus walls (* in Fig. 6C–F). Proximally, therefore, *Mef2c‐AHF‐Cre*‐expressing cells make the greatest contribution to the RΔN and LΔN interleaflet triangles (compare Fig. 6C with D). More distally, NCC made a significant contribution to the commissures, attaching the leaflets to the walls of the sinuses, and also to the luminal side of the RΔN and LΔN interleaflet triangles (compare Fig. 6F with E). Notably, the composition of the RΔL triangle, which interposes between the two aortic sinuses giving rise to the coronary arteries, had a greater contribution from NCC‐derived cells, which are distributed throughout the thickness of the wall (arrowhead in Fig. 6D). As mentioned earlier, the RΔL is the only interleaflet triangle to show expression of Sox9 (see Fig. 5D), indicating its derivation from the fused outflow cushions.

**Figure 8:**
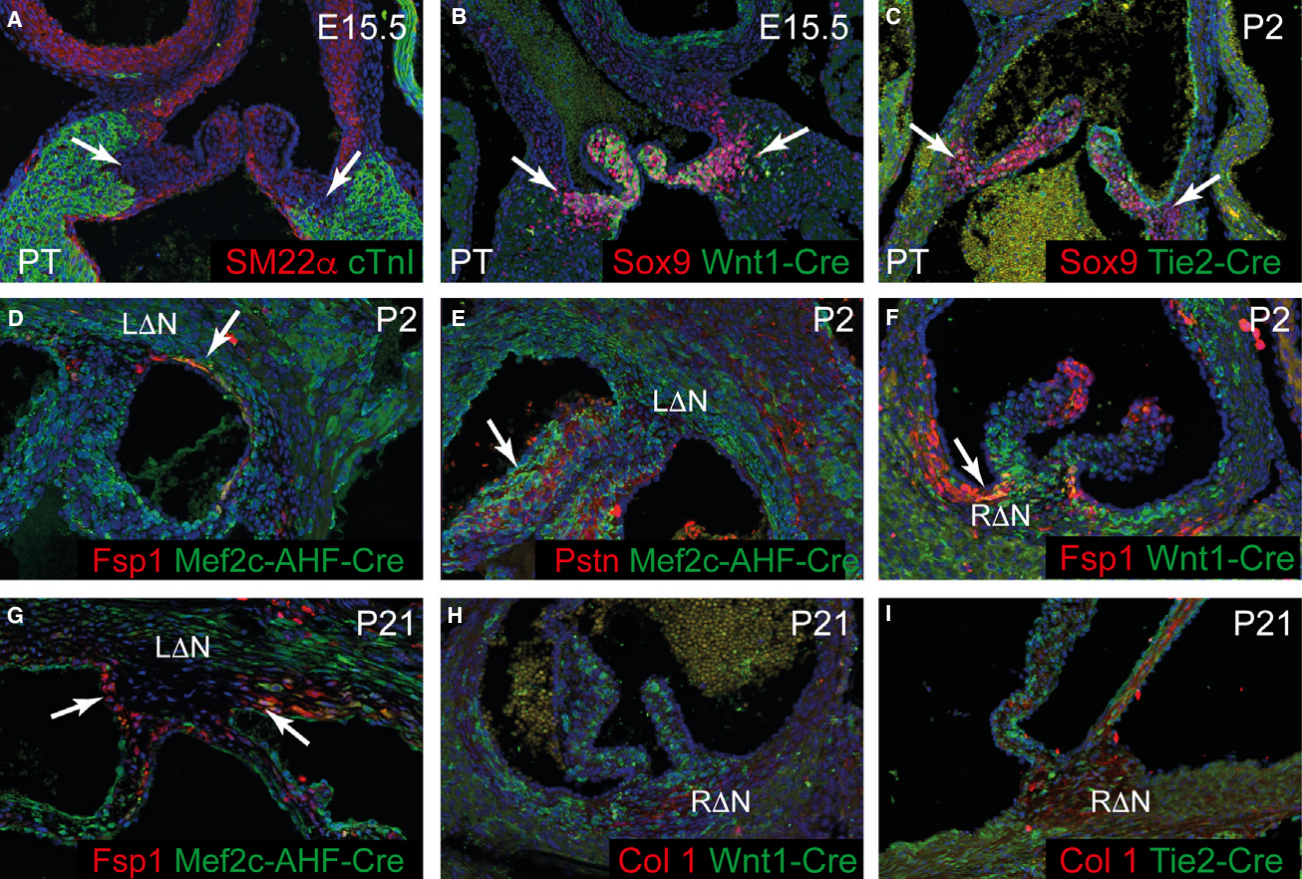
Lineage origins of the fibrous tissues of the mature aortic root. (A–C) The valve hinges are not labelled by Sm22α (red) or cTnI (green) antibodies (A), although they do express Sox9 (red in B and C), which overlaps with both *Wnt1‐Cre* and *Tie2‐Cre* (B,C), supporting the idea that they are derived from the outflow cushions. (D–F) At P2, Fsp1‐expressing cells (red) in the sinus walls and in the lateral regions of the commissures are co‐labelled by *Mef2c‐AHF‐Cre* and *Wnt1‐Cre* (arrows in D, F), suggesting that these are fibroblasts. Periostin does not label these cells, although it is found in the valve leaflets (arrow in E). (G–I) At P21, *Mef2c‐AHF‐Cre, Wnt1‐Cre* and *Tie2‐Cre* label cells within the commissures and interleaflet triangles. Fsp1 is not found within the central parts of these structures, although it is maintained in the lateral regions (arrows in G). Collagen‐I in contrast is found in the central regions of the interleaflet triangles and commissures (H, I). NΔL, left‐non coronary interleaflet triangle (also RΔL); Pstn, periostin.

**Figure 9:**
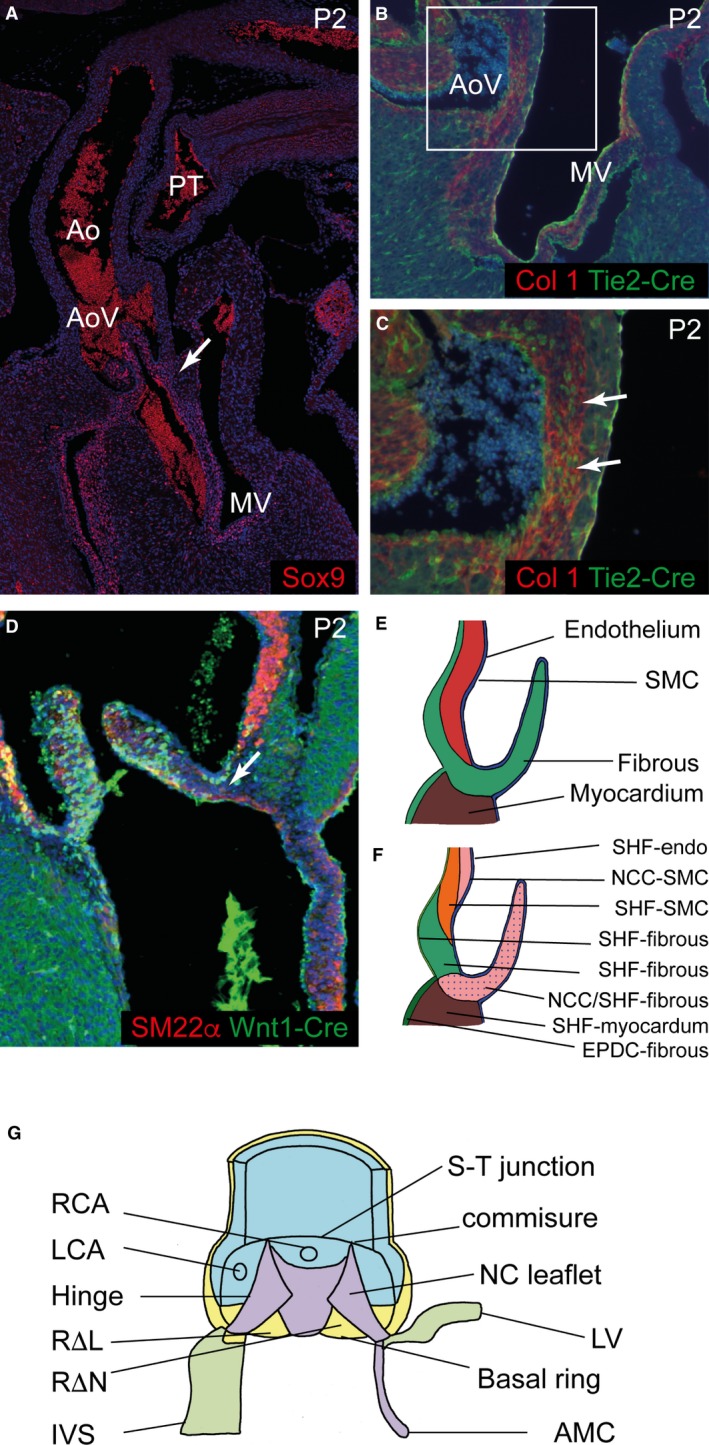
The aortic‐mitral continuity is derived from cushion tissue. (A) The aortic‐mitral continuity expresses Sox9 (red), suggesting it is derived from cushion tissue. (B–D) The aortic‐mitral continuity contains *Tie2‐Cre* labelled cells, but very few *Wnt1‐Cre* labelled cells. (E–G) Cartoons summarising the histological fate of cells in the arterial roots, and their lineage origins. AMC, aortic‐mitral continuity; IVS, interventricular septum; LCA, left coronary artery; LV, left ventricular myocardium; NC leaflet, non‐coronary leaflet; RCA, right coronary artery; RΔL, right‐left interleaflet triangle; RΔN, right‐non‐coronary interleaflet triangle; S‐T junction, sinutubular junction.

At E15.5 and P2, *Wnt1‐Cre* and *Tie2‐Cre* contribute to the valve hinges, which also express Sox9, supporting the notion that they are derived from non‐muscularised cushion tissue (Fig. 8A–C). At the same stages, established cardiac fibroblast markers, including Fsp1 (Fig. 8D–F), were expressed in the interleaflet triangles, and could be seen to co‐localise with both *Mef2c‐AHF‐Cre* (Fig. 8D,E,G) and *Wnt1‐Cre* (Fig. 8F–H) ‐derived cells, as had been shown at earlier stages of development and maturation (Fig. 6). *Tie2‐Cre*‐derived cells did not co‐localise with fibrous markers (Fig. 8I). The aortic‐to‐mitral fibrous continuity, which provides an anatomical bridge between the aortic and mitral valves, also expressed collagen‐I and Sox9, indicating its derivation from cushion tissue (Fig. 9A–C). In contrast to the valve hinges, however, it was composed largely of *Tie2‐Cre*‐expressing cells (arrows in Fig. 9C,D). This area was the original inner curvature of the early pre‐septated heart and hence was initially myocardial. Taken together, these data delineate the origins of the fibrous tissues within the arterial roots. They clarify the relationship between different structures, which may be relevant to disease processes.

## Discussion

In this study we have shown how the aortic and pulmonary roots are developmentally specified mainly from SHF progenitors, and to a lesser degree from NCC. We have clarified how their walls, initially encased exclusively within a myocardial turret, subsequently remodel to form the mature, characteristic pattern of SMC and fibrous tissues within the mature arterialised walls. In this regard, there must also be regression of the initial myocardial walls enclosing the putative fibrous components. Previous studies describing the characteristic appearances of tissues within the arterial roots (Jackson et al. 1995; Ya et al. 1998; Qayyum et al. 2001) have been limited both in terms of incorrect developmental paradigms, and the reagents available to answer key questions. Here, using *Cre‐lox* lineage tracing, we have been able to trace the origins of the cells that form the walls of the arterial roots, demonstrating their eventual differentiation to mature tissues. Specifically, using the *Cre*‐driven recombination of floxed reporter constructs, in conjunction with analysis at consecutive stages of development, we have been able to exclude trans‐differentiation of either SMC or pre‐existing myocardium into the fibrous components of the root. Instead, we have shown their direct origin from SHF and NCC progenitors. Additionally, we show that the hinges and commissures of the arterial valves are developmentally distinct from the walls of their supporting sinuses. They arise, like the valve leaflets, from the outflow cushions. These processes are similar in both pulmonary and aortic roots. Thus, we have provided a framework for understanding malformations and diseases of the arterial roots. Although the development of the valve leaflets themselves is too complex to present within this manuscript, and is the subject of a related study (Eley et al., unpubl. data), it is relevant that the leaflets are also derived from NCC and SHF lineages. Hence, they share origins with the cells formed within the walls of the arterial roots. This provides a direct link between abnormalities of the leaflets and those of the surrounding walls. This could provide a developmental genetic explanation for why dilation of the root can occur in association with abnormalities of the leaflets, such as bicuspid aortic valve.

A longstanding question has been how the arterial valve leaflets come to span the smooth muscle‐myocardial boundary in the mature heart. This can be explained by the finding that the valve hinges, along with the distal attachments of the valves at the commissures, originate together with the leaflets from the outflow cushions. These cushions are originally found within the entire length of the myocardial part of the outflow tract. They form a distinct layer of tissue, filled with extracellular matrix and separated by a basement membrane, only loosely adhering to the outer wall. Thus, these components have the capacity to remodel relative to one another without requiring the dissolution of tight cellular attachments. As cells are added from the SHF to the distal part of the outflow tract to form what will be the intrapericardial components of the arterial trunks, the outflow vessels remodel such that the leaflets are formed within the developing arterial roots. When first recognised, the forming valve complexes are entirely surrounded by outflow tract myocardium, which also supports their basal components. Whether subsequent development reflects a growth of the valve leaflets distally or a remodelling of the base of the leaflets as the sinuses expand, or a combination of both processes, remains to be established. Irrespective of the mechanism, the valve leaflets come eventually to be anchored basally in the ventricular (myocardial) part of the outflow tracts, but are attached distally at the sinutubular junction, which then forms the junction between the roots and the intrapericardial arterial trunks. In this regard, there is also removal of myocardium in the developing aortic root, concomitant with the eventual formation of fibrous continuity between the leaflets of the aortic and mitral valves. The mechanism underpinning this process is the subject of an ongoing investigation in our laboratory.

The interleaflet triangles, which lie within the walls of the arterial root, between the sinuses, have also presented a paradox. Although they lie upstream of the leaflets, and thus are within the ventricle, they are fibrous, and thus have characteristics of arterial tissues. It is important at this point to distinguish the RΔN and LΔN interleaflet triangles from the RΔL. Whereas the RΔN and LΔN triangles are derived from the outflow tract wall, the RΔL is derived from the fused main outflow tract cushions that separate the aorta from the pulmonary trunk. Thus, the fibrous tissue of the RΔN and LΔN interleaflet triangles is derived from the SHF, as are the adjacent myocardium and SMC. In contrast, the RΔL, like the valve leaflets, is composed of both NCC and SHF‐derived cells. Irrespective of their origin, however, and unlike the myocardial and arterial components of the outflow vessels that are distinct by E11.5 (Anderson et al. 2012), the SMC and fibrous components of the arterial roots do not become phenotypically distinct until after birth. Accordingly, the interleaflet triangles are not recognisably fibrous until after birth. Although this has not been specifically examined in human fetuses and newborns, it has been shown that collagen levels increase in the aortic root during the latter half of fetal life (da Fonseca Ferraz et al. 2016). Thus, a similar process of late maturation of the fibrous parts of the aortic root is likely to occur in humans as in mice. It is the semilunar attachment of the valve leaflets that gives the triangles their characteristic shape, with a narrow apex at the commissures and a broad base. Despite being technically within the ventricle, therefore, the triangles are constituents of the arterialised parts of the outflow vessels. What drives this late differentiation remains unclear, although it may be the changes in blood pressure that occur following birth. The difficulties in discriminating between myocardium, SMC, and fibroblasts in a developmental context, in that immature myocardium and motile fibroblasts can express SMC markers such as αSMA and SM22α during developmental stages, make the combination of *Cre‐lox* lineage tracing and careful analysis of serial developmental stages crucial to our study.

It is remarkable that the pulmonary and aortic roots are so similar. Our studies, nonetheless, show small differences between the two that may be of developmental and clinical significance. The asymmetrical levels of NCC in the pulmonary and aortic cushions and sinus walls may be important if there are NCC‐based abnormalities or, conversely, if there are SHF abnormalities. So, for example in congenital aortic stenosis, where the aortic root is narrowed, the genetic causes may lie in the contributions made by the neural crest, which are more evident in the aortic rather than the pulmonary valve. It is notable that, whereas all three interleaflet triangles in the pulmonary root are supported by myocardium, this is not the case in the aortic root, where only the RΔL triangle is entirely supported by the ventricular myocardium. Thus, although the anatomy has been well described in the human heart (Tretter et al. 2016), the embryology has remained obscure. Our clarification of the origins of these tissues may help to understand diseases of the aortic root. The interleaflet triangles are intimately related, both developmentally and anatomically, with the other elements of the root. Indeed, these triangles are abnormal in the setting of bicuspid and unicuspid aortic valves. For example, in bicuspid aortic valve, there are usually two well‐formed interleaflet triangles. If the third triangle exists at all, it is hypoplastic and associated with the raphe in a fused leaflet (Tretter et al. 2016). Similarly, in unicuspid aortic valve, there is usually only one well‐formed interleaflet triangle (Tretter et al. 2016). Both bicuspid and unicuspid aortic valves are associated with aortopathy, which includes dilation of the root or ascending portions of the aorta. The tough fibrous nature of the interleaflet triangles may act to prevent dilation at the base of the valves, in comparison with the walls found more distally, which are dependent on elastin and SMC tone for resisting transmural pressure. Thus, the composition and lineage origins of the different parts of the roots may well have a bearing on the localisation and severity of postnatal pathology. The region of aortic‐to‐mitral fibrous continuity is the remnant of the inner curvature, and sits between the aortic side of the outflow tract cushions and the aortic leaflet of the mitral valve. Although this area is originally myocardial in the primitive looping heart tube, the myocardial component of the wall does not persist. This tissue origin may therefore be important in conditions such as hypoplastic left heart syndrome (HLHS). In that lesion, there may be issues both with the development of the aortic root and its leaflets and the mitral valve in the variant characterised by combined mitral and aortic atresia with a slit‐like left ventricle. In a recent paper (Crucean et al. 2017), we have shown that both the aortic and mitral valve leaflets have contributions from the SHF and from NCC. Our data could be interpreted to support the idea that some subgroups of HLHS result from primary disruption of valve development involving the fibrous components derived from these embryonic cell lineages. Moreover, some sub‐groups of HLHS, where the predominant valvar abnormality involves a small and variably structurally abnormal aortic valve, may have origins in genes expressed in either or both the SHF and NCC components of the aortic root. In this situation the left ventricular hypoplasia may be a secondary effect, perhaps amplified by mutations in other left ventricular myocardial specific genes (Liu et al. 2017). Thus, understanding the development of the outflow tract, specifically its fibrous components, is essential for understanding of the complex architecture of the arterial roots. Such understanding may help in determining the mechanisms by which malformations and diseases come to affect them.

## Author contributions

The study was devised by Deborah Henderson, Bill Chaudhry and Robert Anderson. The experimental work was carried out by Rachel Richardson, Lorraine Eley, Charlotte Donald‐Wilson, Jonathon Davis, Natasha Curley, Ahlam Alqahtani and Lindsay Murphy. The data were analysed and the manuscript was written and revised by Deborah Henderson and Bill Chaudhry, with contributions from Robert Anderson. None of the authors has any conflicts of interest.
